# Giant mass in a 15-year-old girl 

**Published:** 2021

**Authors:** Fatemeh Majidirad, Hossein Asl Soleimani, Anahita Sadeghi, Mohammad-Reza Hadian

**Affiliations:** 1 *Physical Therapy Department, School of Rehabilitation, Tehran University of Medical Sciences, Tehran, Iran*; 2 *Digestive Disease Research Institute (DDRI), Tehran University of Medical Sciences, Tehran, Iran*; 3 *Department of Physical Therapy, School of Rehabilitation, Brain & Spinal Cord Injury Research Center, Institute of Neuroscience, Imam Hospital Complex, Tehran University of Medical Sciences, Tehran, Iran*

## Introduction

**Figure 1:**
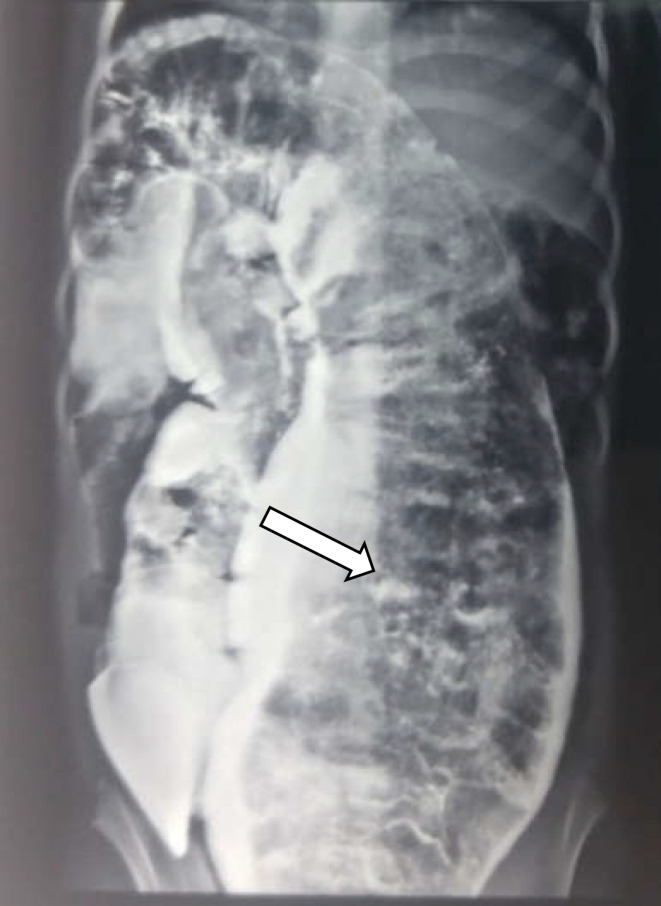
Barium enema showing dilatation of the left colon

**Figure 2:**
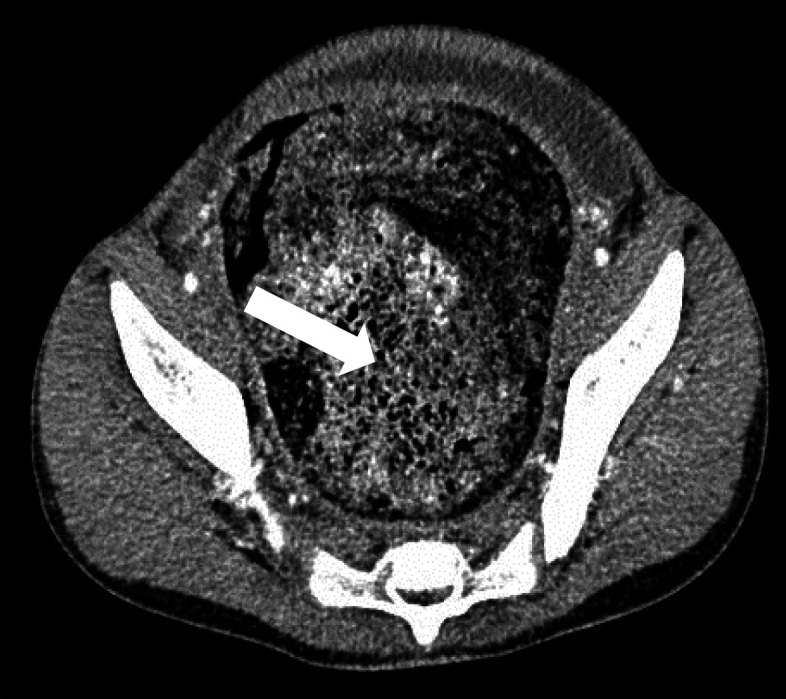
Contrast-enhanced axial CT section showing well-formed, large fecal ball in the dilated rectosigmoid colon

**Figure 3:**
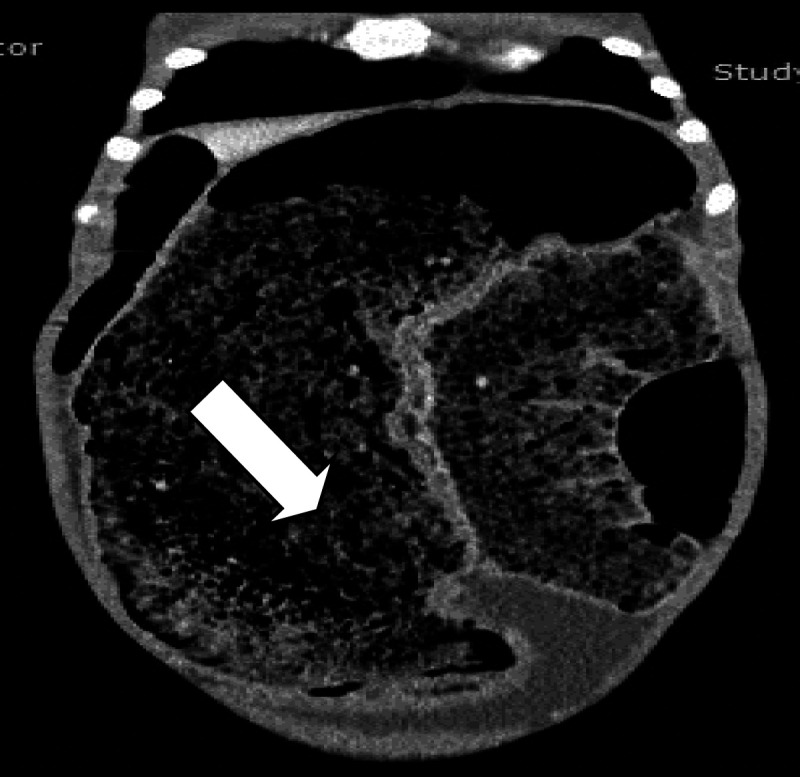
Contrast-enhanced frontal CT section showing extended fecaloma

 A 15-year-old girl referred to a gastroenterology clinic with a chief complaint of passive fecal incontinence once a day in the past two months. Her parents stated that she had had a constipation problem since childhood (i.e. more than 10 years), weight loss, and poor appetite. The frequency of bowel movement was twice per week in a hard, bullet-shaped form (type 1 Bristol stool form scale). The patient always felt incomplete evacuation and used her finger to remove the stool from the rectum. She had been taking laxative medications for several years. No history of abdominal and rectal surgery or previous illness was found in her medical records. On physical examination, the patient was cachectic and underweight (BMI of 16.44) with a dilated and prominent abdomen. A huge, rigid, immobile, non-tender mass in the lower quadrants of the abdomen (especially in the hypogastric region) was palpated. Neurological examination, blood pressure, and temperature were normal. During the *digital rectal exam* (DRE), a significant amount of fecal material was revealed. Laboratory tests revealed WBC 6480 mm3, hemoglobin 12.4 g/dl, hematocrit 39.2%, platelets 363000 mm3, ESR 1st hour 7, creatinine 0.71 mg/dl, and urea 32 mg/dl. Urinalysis, liver, and thyroid function tests were normal. The barium enema of the patient is shown in Figure 1, and CT-scan images are shown in Figures 2 and 3. 

**Diagnosis** **: **Fecaloma due to chronic functional constipation 

Upon examination, a dilated and prominent abdomen was observed, and a huge, rigid, immobile, non-tender mass in the lower quadrants of the abdomen was palpated. During the *digital rectal exam* (DRE), a significant amount of fecal material was revealed. Barium enema showed extensive dilatation of the rectosigmoid and the proximal part of the descending colon. Fecal material was found in the large bowel loops, especially in the distal area. Mucosal lesions were not seen in areas other than stenosis in the distal rectum in favor of Hirschsprung's disease. In the 24-hour evacuation stereotype, the contrast agent was not evacuated (Figure 1).

Computerized tomography (CT) scan with intravenous and oral contrast showed severe distention of the large bowel, which contained a large volume of fecal material, especially in the rectum and sigmoid colon, and the bladder was compressed by a distended rectum. The liver, gallbladder and biliary tree, pancreas, spleen, kidneys, ureters, reproductive organs, stomach, abdominal wall, and bones were normal (Figures 2 and 3).

Abdominal-pelvic ultrasound examination (with a pediatric-specific frequency probe) showed a hyperechogenic mass with poorly defined area measuring about 220 x 90 mm, suggestive of a dilated bowel loop owing to chronic constipation. 

The high-resolution anorectal manometry (HR-ARM) results showed an anal canal length of 3 cm, low maximum resting anal sphincter pressure, low maximum squeezing pressure, abnormal squeeze duration less than 20 seconds, dyssynergia at push maneuver, rectoanal inhibitory reflex (RAIR), normal cough pressure, and normal first rectal sensation of 10 cc. Maximum tolerable volume was not available because of fecal impaction. Eventually, habitual megarectum, dyssynergic defecation, low maximum resting, and squeezing pressure (leading to overflow incontinence) were identified. However, due to the fecal impaction, the result of HR-ARM was doubtful.

Based on the patient’s history, physical and neurological examinations, and the results of imaging and paraclinical tests, organic and neurologic causes of constipation were ruled out (1). Moreover, the presence of RAIR excluded the possibility of Hirschsprung’s disease (2). Consequently, fecaloma because of chronic functional constipation was diagnosed due to the stool withholding behavior.

Before any treatment, the fecaloma had to be evacuated. The pulsed irrigation evacuation (PIE) method was advised to parents, but they rejected it due to its high cost and lack of coverage by the insurance company. Therefore, a simple and less expensive method of lactulose enema was explained to the parents and the patient, and a written consent form was signed. The solution of lactulose syrup (250 cc) and warm water (750 cc) was injected into the anus by enema bulb rectal syringe while the patient was lying on her left side (3). The procedure was taught to the patient’s parents and was performed twice daily. The procedure was continued as long as the patient could tolerate it, and her parents were asked to monitor the patient's pulse rate and respiratory rhythm and to seek medical care if problems occurred. The patient evacuated a massive and soft stool after each producer; in three weeks follow up, there was no abdominal mass and palpation of the abdomen was completely normal. 

Furthermore, nutritional counseling and the use of a high-fiber diet and plenty of fluids were recommended. Osmotic laxatives (i.e. polyethylene glycol [PEG]) and glycerin suppositories were also administered. Continuing treatment with lactulose enemas was emphasized to the parents, if the patient did not have a normal pattern of defecation. The patient was also instructed in diaphragmatic breathing. Then, she was referred to psychological counseling and a physiotherapist for behavioral therapy, strengthening of the pelvic floor and abdominal muscles, relaxation techniques, and toilet posturing. After 2 years of follow-up by telephone, the patient’s parents reported they did not refer to psychological and physiotherapy counseling, but the patient had experienced no complications, had a defecation frequency of two or three times per week, and returned to school.

This was a rare case of giant fecaloma due to stool withholding behavior which had not been reported previously. Fecaloma can form a large, firm, relatively immobile tumor-like mass in the abdomen (4, 5). Factors that can cause fecalomas include Hirschsprung's disease, Chagas disease, and chronic constipation. It can also be seen in patients with movement disorders, like in neurological disease (spinal cord injuries, Parkinson's, multiple sclerosis) and musculoskeletal patients (bedridden patients, patients after orthopedic problems) and patients with psychiatric problems as well as those with dementia, Alzheimer's, or endocrine diseases. Fecaloma can also be caused by medications such as diuretics, narcotics, and calcium channel blockers. In children, slow transit constipation, dyssynergia, and stool withholding behavior should also be considered (4, 6-10). 

Fecaloma causes several complications such as ischemia in the intestinal wall and the onset of inflammation. Eventually, it can ulcerate and perforate the intestine or cause intestinal obstruction and megarectum. It can also compress adjacent structures, especially the bladder, and even cause its perforation; obstruction of the urethra; pyelonephritis; and rectovaginal fistula. Abdominal compression syndrome (ACS) can be another complication with a high rate of mortality (7, 11-13). Therefore, early treatment of constipation and its follow-up is very important. Fecaloma can be managed conservatively with laxatives, enemas, and digital disimpaction. In more severe a or even life-threatening cases, more invasive procedures such as endoscopy or surgery are employed (12, 14). 

In the current case, the patient had adapted herself to accumulate feces over a long time, and she had no complaints other than passive fecal incontinence, weight loss, and poor appetite. The patient was treated with laxatives and suppositories whereas she had stool withholding behavior. Ignoring the defecatory urge may be an unconscious automatic habit of the child resulting from changed or reduced brain processing of urge sensations owing to loss of attention or an intentional decision due to the lack of a private lavatory or a history of painful evacuation (10). As this is a habitual problem, it should be treated with psychological counselling and behavioral therapy alongside other conservative treatments (9). For the management of constipation in these patients, lifestyle modification and the use of laxatives and enemas, psychological counselling, and behavioral therapy should be considered.
